# Elevated serum urea-to-creatinine ratio is associated with adverse inpatient clinical outcomes in non-end stage chronic kidney disease

**DOI:** 10.1038/s41598-022-25254-7

**Published:** 2022-12-02

**Authors:** Elizabeth M. Brookes, David A. Power

**Affiliations:** 1 Melbourne Medical School, The University of Melbourne, Parkville, VIC Australia; 2 The Department of Nephrology, Austin Health, Heidelberg, VIC Australia; 3 The Department of Medicine, The University of Melbourne, Victoria, Australia

**Keywords:** Kidney, Epidemiology

## Abstract

To better understand the role of the urea-to-creatinine ratio in chronic kidney disease patients, we assessed the epidemiology of the urea-to-creatinine ratio among hospitalised chronic kidney disease patients, and the association between the urea-to-creatinine ratio and inpatient clinical outcomes. This retrospective cohort study (*n* = 11,156) included patients with at least two eGFR values < 60 mL/min/1.73m^2^ measured greater than 90-days apart and admitted to a tertiary hospital between 2014 and 2019. Dialysis and renal transplant patients were excluded. Adjusted odds ratios for factors associated with an elevated urea-to-creatinine ratio were calculated. Multivariate regression was conducted to identify the relationship between elevated UCR and inpatient mortality, intensive care admission, hospital readmission and hospital length-of-stay. Urea-to-creatinine ratio > 100 was present in 27.67% of hospital admissions. Age ≥ 65 years, female gender, gastrointestinal tract bleeding, heart failure, acute kidney injury and lower serum albumin were associated with elevated urea-to-creatinine ratio. Higher urea-to-creatinine ratio level was associated with greater rates of inpatient mortality, hospital readmission within 30-days and longer hospital length-of-stay. Despite this, there was no statistically significant association between higher urea-to-creatinine ratio and intensive care unit admission. Elevated urea-to-creatinine ratio is associated with poor clinical outcomes in chronic kidney disease inpatients. This warrants further investigation to understand the pathophysiological basis for this relationship and to identify effective interventions.

## Introduction

The biological role of urea in chronic kidney disease (CKD) remains contentious. Urea has traditionally been thought to be a relatively inert molecule, however, recent experimental data has suggested that it induces biochemical alterations with a potential impact on clinical outcomes^1^. Due to their small molecular sizes, both creatinine and urea are filtered by the glomerulus^2^. Creatinine is not reabsorbed and is excreted from the body, however, approximately 40–50% of urea is reabsorbed in the tubules, where it is linked to reabsorption of sodium and water^3^. Because this process is regulated by both neurohormonal activity and renal function, the urea-to-creatinine ratio (UCR) has been proposed to be of value in clinical practice. In renal failure, serum urea and creatinine levels usually rise proportionally with a progressive decline in renal function^4–6^. Serum urea levels can be further increased by excess protein intake, hypovolaemia, heart failure, gastrointestinal bleeding and catabolism^7^. Increases in serum urea out of proportion to serum creatinine result in an elevated UCR and reflect a critical condition.

Over the past decade, UCR has been strongly associated with poor clinical outcomes in various population settings, such as acute kidney injury (AKI)^8,9^, acute decompensated heart failure^3,10–14^, chronic heart failure^15–17^, acute myocardial infarction^18,19^ and ischaemic stroke^20^. Several studies in haemodialysis patients have shown that UCR level is significantly associated with an increased risk for all-cause mortality^21,22^, infection-related death and incidence of coronary heart disease^23^. A more recent multicentre prospective study from Japan, demonstrated that UCR level at the time of dialysis initiation is associated with all-cause mortality, and is more strongly associated with mortality than eGFR or creatinine clearance alone^7^. The association between UCR and clinical outcomes in non-end stage CKD patients remains uncertain, however, with no current studies in this patient population.

The present study aims to examine the prevalence of elevated UCR in a tertiary hospital-based cohort of CKD patients, as well as its associated patient characteristics and clinical outcomes. We hypothesised that elevated UCR would be associated with poorer inpatient clinical endpoints.

## Method

### Study design & source population

This retrospective observational cohort study used Austin Health electronic medical record data extracted from The Data Analytics Research and Evaluation (DARE) Centre as outlined in a previous study^24^.

### Identification of the study cohort

**Figure 1:**
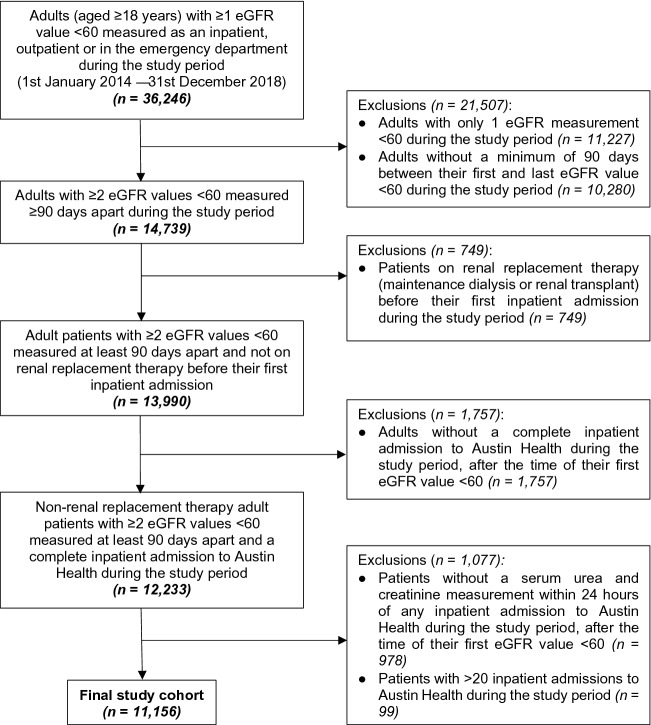
Patient selection for the study cohort. The study cohort included adult patients (aged ≥ 18 years) with at least two abnormal eGFR values (< 60 mL/min/1.73m^2^) measured greater than 90-days apart between 1st January 2014 and 31st December 2018 in inpatient, outpatient or emergency department settings. Adult patients were included if they had a serum urea and creatinine tests performed within 24-h of any inpatient admission to Austin Health following their first abnormal eGFR result. Patients with end-stage kidney disease, defined as maintenance dialysis (both haemodialysis and peritoneal dialysis) or renal transplant, were excluded from the time of the procedure. Patients with greater than 20 inpatient admissions were also excluded. Abbreviations: eGFR, estimated Glomerular Filtration Rate measured in mL/min/1.73m^2^.

The study population included adult patients (aged ≥ 18 years) with at least two abnormal estimated glomerular filtration rate (eGFR) values (< 60 mL/min/1.73m^2^) measured greater than 90-days apart between 1 January 2014 and 31 December 2018 in inpatient, outpatient or emergency department settings at Austin Health (Fig. 1)^24^. Patients were included if they had serum urea and serum creatinine tests performed within 24-h of any inpatient admission following their first abnormal eGFR result. Only inpatient serum urea and serum creatinine results were used for analysis to ensure that sufficient patient information was available (e.g. comorbidities and medications) because electronic medical records are not currently used in the outpatient setting at Austin Health. Patients with greater than 20 inpatient admissions during the study period were removed from the dataset as outliers. Patients with end-stage kidney disease (ESKD), defined as maintenance dialysis (haemodialysis or peritoneal dialysis) or renal transplant, were excluded from the time of the procedure. ESKD was identified by the International Classification of Diseases, Tenth Revision (ICD-10 Australian Modification) code assignment and from comparison with the Austin Health Department of Nephrology Renal Replacement Therapy Database.

### Patient characteristics

Sociodemographic information including age, sex and Indigenous status was reported for patients who were stratified by admission serum UCR level at their first inpatient admission during the study period (Table 1). An UCR level > 100 was used as the cut off for elevated UCR in keeping with previous studies^4,5,8^. Additionally, admission eGFR, comorbidities, the principal diagnosis of the admission, laboratory values, and medications administered during the inpatient episode were recorded.

**Table 1:** Baseline characteristics of the study population.

	Overall cohort (*n* = 11,156)		UCR ≤ 100 (*n* = 8,518)		UCR > 100 (*n* = 2,638)		*p* value
Age (years)	77.30	$\pm$12.03	76.28	$\pm$ 12.26	80.58	$\pm$ 10.60	0.001*
	79	(71–86)	79	(70–85)	83	(76–88)	
Male	5482	(49.15%)	4553	(53.45%)	929	(35.22%)	< 0.001*
ATSI	36	(0.32%)	31	(0.36%)	5	(0.19%)	0.293
eGFR (mL/min per 1.73m^2^)	45.77	$\pm$ 16.55	46.05	$\pm$ 16.60	44.88	$\pm$ 16.34	0.001*
GIT Bleeding	258	(2.31%)	133	(1.56%)	125	(4.74%)	< 0.001*
Cardiac failure	1313	(11.77%)	895	(10.51%)	418	(15.85%)	< 0.001*
Hypovolaemia	385	(3.45%)	267	(3.13%)	118	(4.47%)	0.001*
Coronary artery disease	313	(2.81%)	231	(2.71%)	82	(3.11%)	0.281
Cerebrovascular disease	416	(3.73%)	352	(4.13%)	64	(2.43%)	< 0.001*
Diabetes mellitus	3566	(31.96%)	2666	(31.30%)	900	(34.12%)	0.007*
CCI score excluding age	1.57	$\pm$ 1.85	1.53	$\pm$ 1.83	1.68	$\pm$ 1.92	0.001*
Serum creatinine ($\mu$ mol/L)	136.31	$\pm$ 93.76	139.72	$\pm$ 102.92	125.27	$\pm$ 53.07	< 0.001*
Serum urea (mmol/L)	11.15	$\pm$ 6.53	9.76	$\pm$ 5.57	15.64	$\pm$ 7.32	< 0.001*
Haemoglobin (g/L)	122.97	$\pm$ 21.38	124.41	$\pm$ 21.29	118.34	$\pm$ 21.02	< 0.001*
Potassium (mmol/L)	4.53	$\pm$ 0.63	4.49	$\pm$ 0.61	4.64	$\pm$ 0.68	< 0.001*
Bicarbonate (mmol/L)	23.68	$\pm$ 3.71	23.71	$\pm$ 3.60	23.59	$\pm$ 4.03	0.030*
Sodium (mmol/L)	138.67	$\pm$ 4.26	138.64	$\pm$ 4.20	138.75	$\pm$ 4.47	0.239
Albumin (g/L)	33.75	$\pm$ 5.28	33.86	$\pm$ 5.27	33.40	$\pm$ 5.31	< 0.001*
ACEi/ARB	3860	(34.60%)	2943	(34.55%)	917	(34.76%)	0.842
Beta blocker	3709	(33.25%)	2762	(32.43%)	947	(35.90%)	0.001*
Calcium channel blocker	2380	(21.33%)	1852	(21.74%)	528	(20.02%)	0.058*
Loop diuretic	3487	(31.26%)	2446	(28.72%)	1041	(39.46%)	< 0.001*
Thiazide diuretic	1135	(10.17%)	785	(9.22%)	350	(13.27%)	< 0.001*

*UCR* Urea-to-Creatinine Ration; *ATSI* Aboriginal or Torres Strait Islander; *eGFR* Estimated Glomerular Filtration Rate; *GIT* Gastrointestinal Tract; *CCI* Charlson Comorbidity Index; *ACEi* Angiotensin-Converting Enzyme Inhibitor; *ARB* Angiotensin II Receptor Blocker;

* Patient characteristics with a *p* value < 0.05 are significantly different between patient groups.

#### Definition of chronic kidney disease

Although many of the participants were not formally diagnosed with CKD by a clinician, we defined CKD as at least two eGFR values < 60 mL/min/1.73m^2^ measured greater than 90-days apart during the study period in inpatient, outpatient or emergency department settings. This definition was used to reduce the likelihood of including patients suffering from an AKI in the setting of normal baseline renal function rather than underlying CKD. Patients with an acute on chronic kidney injury were included. eGFR was calculated from serum creatinine levels using the Chronic Kidney Disease Epidemiological Collaboration (CKD-EPI) equation^25^. Kidney Disease Improving Global Outcomes (KDIGO) CKD stages were not reported because data on albuminuria and structural or pathological renal abnormalities was not available^26^.

#### Serum urea-to-creatinine ratio classification

The admission serum UCR was defined as the ratio of the first concomitant serum urea and serum creatinine value taken within 24-h of inpatient admission, inclusive of investigations performed in the emergency department. A UCR level > 100 was used as the cut off for elevated UCR in patient characteristic analyses, in keeping with previous studies^4,5,8^. In the setting of AKI a UCR level > 100 has traditionally been thought to suggest a pre-renal pathology, however, threshold values in other clinical settings have not been extensively investigated or established. For this reason, admission UCR was also categorised into four quartiles (9.98–67.00, 67.01–83.12, 83.13–103.23, ≥ 103.24). The lowest quartile was selected as the reference group for outcome comparison based on clinical relevance and previous reports^7^. To convert SI-units to conventional units (e.g. blood urea nitrogen and creatinine in mg/dL) the UCR should be divided by four^9^.

#### Identification of comorbidities, principal diagnoses & medications

Patient comorbidities, including myocardial infarction, heart failure, cerebrovascular disease and diabetes were based on the Charlson Comorbidity Index (CCI) at admission and grouped by pre-defined ICD-10 codes according to the method outlined by Quan et al.^27^. Important admission diagnoses and complications, such as AKI, gastrointestinal (GIT) bleeding and hypovolaemia, were also determined by ICD-10 codes assigned to pre-defined diagnosis and complication groups. Patient medications were recorded if the individual was administered a medication of interest at any time during their admission. Outpatient medication prescription information was not readily available for analysis. Medications of interest included angiotensin converting enzyme inhibitors (ACEi), angiotensin II receptor blockers (ARB), aldosterone antagonists, *β*-blockers, calcium-channel blockers, loop diuretics and thiazide diuretics. Patients taking prescription medications containing one or more drug classes were credited with receiving both corresponding medications.

### Clinical outcomes

The principle clinical outcome of this study was inpatient mortality. Inpatient mortality was defined as patient death of any cause during the hospital admission episode. Secondary outcomes included hospital readmission within 30-days, hospital length-of-stay, and intensive care unit (ICU) admission. Hospital readmission within 30-days was defined as readmission to the tertiary study centre within 30-days of discharge from the facility, excluding patients who died during admission. Hospital length of-stay was the total length of the admission reported in days, and admission to intensive care was classified as any length of time where patient care was provided in the intensive care unit.

### Data handling & statistical analysis

All data handling and statistical analyses were performed using Microsoft® Excel (Office 365, 2019) and Stata/IC 15.1 (StataCorp, TX, USA). Continuous variables are described as mean ± 1 standard deviation (SD), whereas categorical variables are described as frequencies and percentages. Baseline patient characteristics were compared between patients with their first admission UCR within the normal range and patients with elevated UCR (UCR > 100) using the Kruskal–Wallis H test for continuous variables and the Chi-square test for categorical variables. Logistic regression analyses were performed in the total study population to assess the association of various demographic and clinical characteristics with elevated UCR. The inclusion of variables in univariate analysis was based on existing knowledge of UCR abnormalities in general patient populations. These include age, gender, eGFR, gastrointestinal (GIT) bleeding, cardiac failure, hypovolaemia, AKI, albumin level and use of diuretics. Variables were tested for interaction and included in the multivariate model if *p* < 0.01 in univariate analysis. We report unadjusted and adjusted Odds Ratios (OR) with 95% Confidence Intervals (95% CI). Two-tailed probability values of *p* < 0.05 were considered statistically significant in the final multivariate models. We also performed multivariate logistic regression analyses across UCR quartile groups to evaluate for independent associations between admission UCR level and all-cause inpatient mortality, readmission to hospital within 30-days of discharge and ICU admission, and linear regression for hospital length-of-stay. These models were adjusted for age, sex, eGFR, AKI and comorbidities accounted for in the Charlson Comorbidity Index^24^.

### Ethics and consent statement

This research complies with the guidelines for human studies and was conducted ethically in accordance with the World Medical Association Declaration of Helsinki. This study was approved by the Austin Human Research Ethics Committee and the Austin Health Office for Research (HREC-43815-Austin-2018 and Audit/20/Austin/139). As this study used data routinely collected in healthcare, The Austin Hospital at Austin Health waived the need to obtain informed consent from the participants.

## Results

### Study population

36,246 adults with at least one abnormal eGFR < 60 mL/min/1.73m^2^ during the study period were identified^24^. In an attempt to prevent inclusion of patients with an AKI on a background of normal baseline renal function, 11,227 of these patients were excluded from the study because they only had the one abnormal eGFR measurement, as were a further 10,280 patients who did not have a minimum of 90-days between their first and last abnormal eGFR value. From the 14,739 participants who met the eGFR inclusion criteria, 749 patients with ESKD (haemodialysis or renal transplant) were removed. 1,757 patients did not have an inpatient admission during the study period and 978 patients did not have an available serum urea and serum creatinine measurement within 24-h of an inpatient admission. A further 99 patients were excluded from the study because they had greater than 20 Austin Health inpatient admissions during the study period as outliers. This resulted in a final sample of 11,156 participants (Fig. 1).

### Baseline descriptive data

The study population comprised 11,156 participants with a mean age of 77.30 ± 12.03 years; 49.15% were male and 0.32% identified as Aboriginal or Torres Strait Islander^24^. The mean eGFR was 45.77 ± 16.55 mL/min/1.73m^2^. Demographic and clinical characteristics of patients were significantly different across UCR categories (Table 1). In total there were 38,730 admissions during the study period, with a median of 5 admissions (IQR 3–8) per patient over 5 years.

### The distribution of UCR within the population

**Figure 2:**
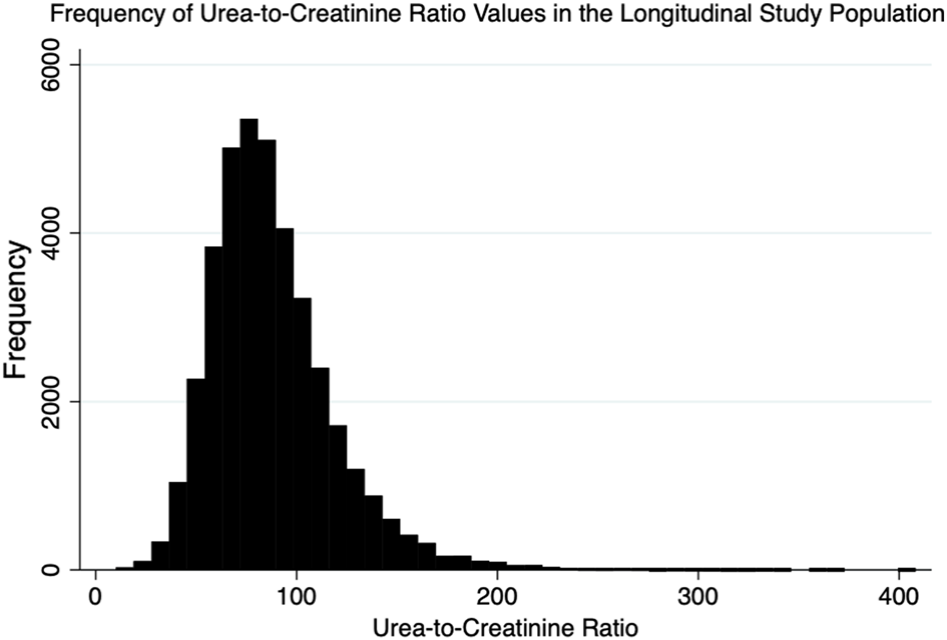
Distribution of admission UCR values. The mean admission serum urea-to-creatinine ratio in the population studied was 88.06 ($\pm$ 31.46). The median admission serum urea-to-creatinine ratio was 83.13 (IQR 67.00–103.23). The minimum admission urea-to-creatinine ratio was 9.98, and the maximum urea-to-creatinine ratio was 408.26.

The mean admission serum UCR was 88.06 ± 31.46 over the study period (Fig. 2). The minimum serum UCR value was 9.98, and the maximum serum UCR value was 408.26. The prevalence of an elevated UCR at admission (UCR > 100) was 27.67%. Moreover, 4.10% of admission UCR values were > 150.

### Patient characteristics associated with elevated UCR

The results of the univariate and multivariate logistic regression analyses for patient characteristics associated with elevated serum UCR in the study cohort appear in Table 2. Age ≥ 65 years, GIT bleeding, cardiac failure, AKI, greater comorbidity burden and diuretic use showed significantly elevated OR for the development of an elevated serum UCR in both univariate and multivariate analyses. Male sex, declining renal function and higher serum albumin level was associated with lower OR of elevated UCR. There was no statistically significant relationship established between hypovolaemia and UCR.

**Table 2:** Patient characteristics associated with elevated urea-to-creatinine ratio (UCR > 100).

	Univariate analysis			Multivariate analysis		
	Odds ratio	95% CI	*p* value	Odds ratio	95% CI	*p* value
Age ≥ 65 years	2.21	(1.95–2.50)	< 0.001*	2.04	(1.79–2.33)	< 0.001**
Male versus female	0.54	(0.50–0.58)	< 0.001*	0.56	(0.52–0.61)	< 0.001**
eGFR (per 5 mL/min per 1.73m^2^ reduction in eGFR)	1.02	(1.01–1.04)	< 0.001*	0.95	(0.93–0.96)	< 0.001**
Albumin	0.98	(0.98–0.99)	< 0.001*	0.99	(0.98–0.99)	< 0.001**
GIT bleeding	2.17	(1.88–2.50)	< 0.001*	2.27	(1.94–2.67)	< 0.001**
Cardiac failure	1.79	(1.62–1.98)	< 0.001*	1.36	(1.24–1.48)	< 0.001**
Hypovolaemia	1.27	(1.12–1.44)	< 0.001*	1.11	(0.96–1.27)	0.154
Acute kidney injury	1.39	(1.31–1.48)	< 0.001*	1.49	(1.37–1.62)	< 0.001**
CCI score Excluding age	1.06	(1.04–1.07)	< 0.001*	1.03	(1.01–1.05)	0.001**
Loop diuretic	1.84	(1.73–1.95)	< 0.001*	1.53	(1.43–1.65)	< 0.001**
Thiazide diuretic	1.55	(1.40–1.71)	< 0.001*	1.50	(1.35–1.67)	< 0.001**

95% *CI* 95% Confidence interval; *eGFR* Estimated glomerular filtration rate; *GIT* Gastrointestinal tract; *CCI* Charlson comorbidity index.

* Patient characteristics with a *p* value < 0.01 in univariate analysis were selected for inclusion in multivariate analysis.

** *p* value < 0.05 in multivariate analysis was considered statistically significant.

### Clinical outcomes

#### All-cause inpatient mortality

**Figure 3:**
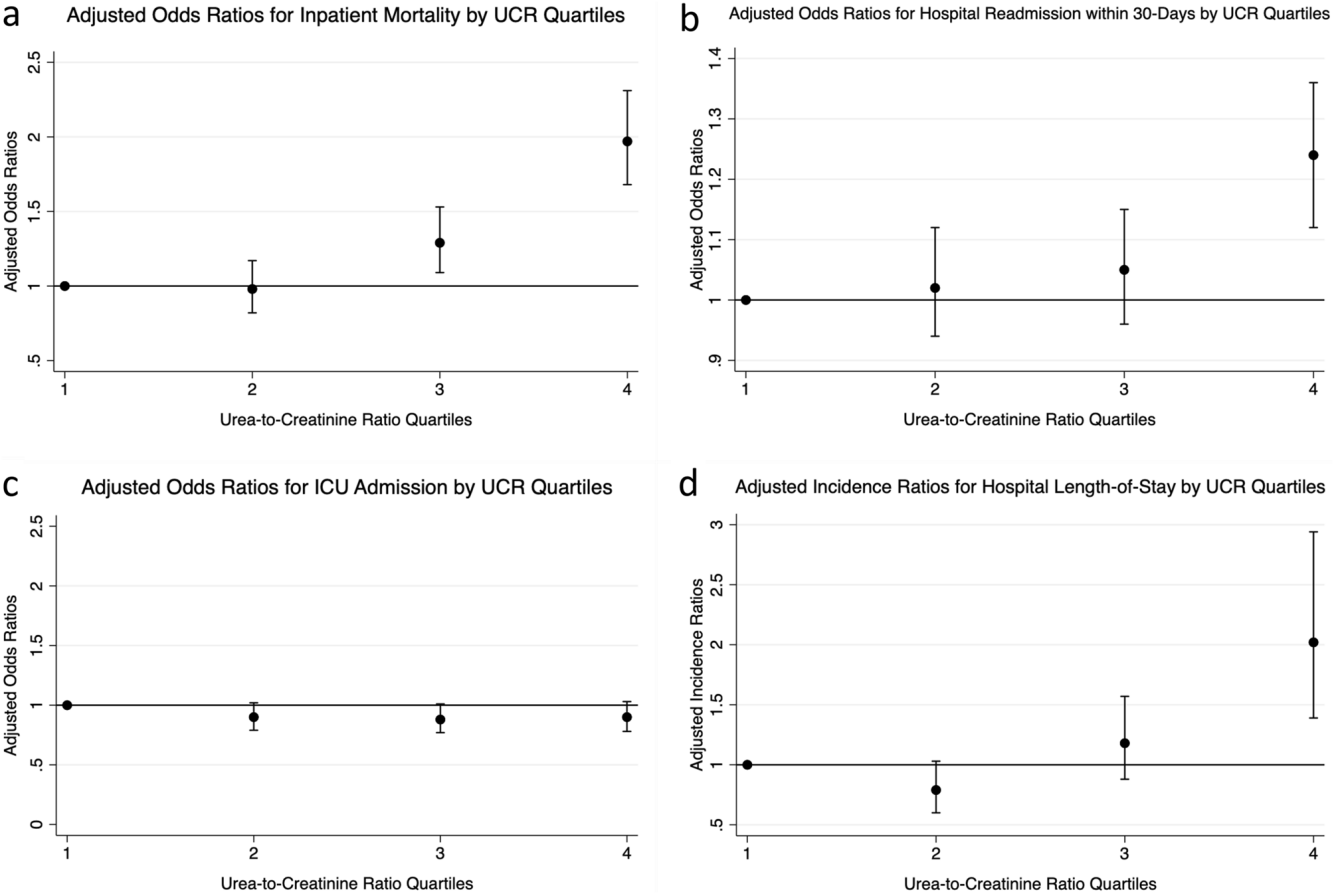
Clinical Outcomes by UCR Quartile. All graphs show adjusted odds ratios (or incidence ratios in the case of hospital length-of-stay) with 95% confidence intervals from multivariate analysis for the clinical outcomes of interest by admission UCR quartile, represented by a dot with error bars. The dotted grey line represents an OR of 1.00. Data points with 95% confidence intervals that cross the grey line are not considered statistically significant. Models are adjusted for age, sex, CCI score, eGFR, GIT bleed, heart failure and hypovolaemia. (**a**): Adjusted Odds Ratios for Inpatient Mortality by UCR Quartile. (**b**): Adjusted Odds Ratios for Readmission to Hospital within 30-Days by UCR Quartile. (**c**): Adjusted Odds Ratios for ICU Admission by UCR Quartile. (**d**): Adjusted Incidence Ratios for Hospital Length-of-Stay by UCR Quartile.

Among the study population, 1,659 (4.28%) patients died during an inpatient admission throughout the 5-year study period. 43.76% of these inpatient deaths occurred in patients with an admission UCR > 100. Patients in the 3rd and 4th quartiles for UCR had a dose-dependent statistically significant increased risk of all-cause inpatient mortality with OR 1.29 (95% CI 1.09–1.53, *p* = 0.003) and OR 1.97 (95% CI 1.68–2.31, *p* < 0.001), respectively (Table 3, Fig. 3a).

**Table 3:** Clinical outcomes associated with urea-to-creatinine ratio level.

UCR quartile	Adjusted OR	95% CI	*p* value
**Inpatient mortality**			
Quartile 2	0.98	(0.82–1.17)	0.804
Quartile 3	1.29	(1.09–1.53)	0.003*
Quartile 4	1.97	(1.68–2.31)	< 0.001*
**Hospital readmission within 30-days**			
Quartile 2	1.02	(0.94–1.12)	0.631
Quartile 3	1.05	(0.96–1.15)	0.255
Quartile 4	1.24	(1.12–1.36)	< 0.001*
**Intensive care unit admission**			
Quartile 2	0.90	(0.79–1.02)	0.087
Quartile 3	0.88	(0.77–1.01)	0.066
Quartile 4	0.90	(0.78–1.03)	0.122
**Hospital length-of-stay**			
Quartile 2	0.79	(0.60–1.03)	0.076
Quartile 3	1.18	(0.88–1.57)	0.269
Quartile 4	2.02	(1.39–2.94)	< 0.001*

*UCR* Urea-to-creatinine ratio; *OR* Odds ratio; 95% *CI* 95% Confidence interval; *IR* incidence ratio.

Odds/incidence ratios were adjusted for age, sex, CCI score, eGFR and acute kidney injury status on admission by multivariate logistic regression analysis.

*p value < 0.05 in multivariate analysis was considered statistically significant.

#### Hospital readmission within 30-days

In the total study population, 5,791 (14.95%) eligible hospital discharges (excluding mortality) resulted in readmission to Austin Health within 30-days. 1,740 (30.05%) of these readmissions occurred in patients with an elevated UCR > 100. In multivariate analysis, only patients with an admission UCR in the 4th quartile had a statistically significant increased risk of 30-day readmission with OR 1.24 (95% CI 1.12–1.36, *p* < 0.001) (Table 3, Fig. 3b).

#### Intensive care admission

2,175 (5.62%) patient admissions required ICU admission, but only 555 (25.52%) of these admissions occurred in patients with an elevated UCR. Despite the previously noted increased risk of mortality, no statistically significant trend in ICU admissions was found across UCR quartiles. 689 (7.11%) patients in the 1st quartile, 512 (5.30%) patients in the 2nd quartile, 476 (4.91%) patients in the 3rd quartile, and 498 (5.14%) patients in the 4th quartile required ICU admission(Table 3, Fig. 3c).

#### Hospital length-of-stay

The median hospital length of stay in the total population was 3 days (IQR 1–9 days). The median hospital length of stay in patients with UCR > 100 was 4 days (IQR 1–11 days) compared to 3 days (IQR 1–8 days) in patients with normal UCR. This represented an IR of 2.02 (95% CI 1.39–2.94, *p* < 0.001) in patients with their admission UCR values in the 4th quartile (Table 3, Fig. 3d).

## Discussion

Elevated UCR is common in patients with CKD, as 27.67% of CKD inpatients had a UCR > 100 at the time of admission. The mean admission serum UCR was 88.06 ± 31.46 over the study period, which is similar to other recent observational studies in different patient population groups^4,8,14,15^.

It has been proposed that increased UCR may be a useful biomarker indicative of patient frailty^9^. Multiple factors that are associated with frailty can mechanistically result in an elevated UCR. Firstly, the elderly are typically more susceptible to dehydration due to decreased thirst sensation, reduced mobility as a barrier to hydration, comorbidities and diuretic use^28^. Indeed, in the present cohort, non-end stage CKD patients with a higher UCR were generally older with concurrent, cardiac failure, diuretic use and a greater comorbidity burden. Heart failure, AKI, diuretic use and dehydration result in an elevated UCR by increasing reabsorption of urea in the renal tubules due to a decrease in glomerular filtration rate^4,7,13^. In the present study, hypovolaemia was not found to be statistically significant in multivariate analysis, however, this may have been because of the variability in recording this subjective measure in the clinical record. Furthermore, frail patients are more likely to suffer from reduced serum creatinine levels secondary to low muscle mass and sarcopenia, thus increasing their UCR^29^. Unfortunately, body mass index (BMI) data was not available for analysis in our population, however, serum albumin may act as a surrogate marker of nutrition and lower serum albumin levels correlated with a higher UCR in this study. Lastly, in the study cohort, GIT bleeding, which may increase catabolism and can increase the absorption of urea from the intestines, was associated with elevated UCR^7^. Of note, our study was limited in that the data on dietary intake, specifically that of protein, was not available for analysis and high-protein diets are likely to enhance urea production^13^. Overall, however, these results demonstrate that the patient factors associated with elevated UCR in this non-end stage CKD population are consistent with previous studies in other population groups and are biologically plausible.

This study showed a clear association between elevated UCR and an increased risk for all-cause inpatient mortality, increased hospital length-of-stay, and readmission within 30-days. The associations remained even after adjusting for potential confounders in multivariate analysis. These findings are in keeping with previous studies in ESKD patients. In a prospective study of 3,401 maintenance haemodialysis patients in Japan, for example, Tanaka et al. determined that every 1 increase in UCR level was significantly associated with an increased risk for all-cause mortality, coronary heart disease, and infection-related death^23^. Two further studies found that elevated UCR at the time of initiation of dialysis was independently associated with early mortality and all-cause mortality^7,21^. Furthermore, UCR at initiation of dialysis had a stronger association with these outcomes than other factors including eGFR, or urea or creatinine alone. Results from the current study, therefore, support existing literature and extend the correlation between elevated urea-to-creatinine ratio and all-cause inpatient into the area of non-end-stage CKD.

One curious observation is that there was no statistically significant association between ICU admission and an elevated UCR, despite the association between elevated UCR other adverse clinical outcomes. Sub-analysis by age group, comorbidities and renal function failed to reveal a statistically significant trend. This observation may reflect the adoption of therapies designed to keep patients out of ICU, such as high protein feeding, but this is speculative. An alternative explanation may be that because the patients who are most at risk of an elevated UCR are an older and more comorbid population, their goals of care may exclude admission to ICU, however, advanced resuscitation plan data was not available for analysis in this study.

The pathophysiological mechanism underpinning the association between elevated UCR and adverse clinical outcomes remains unclear. Classically, urea has been thought to be a relatively inert molecule, however, recent experimental studies indicate that at serum concentrations typical of CKD, urea interferes both directly and indirectly with several important biochemical functions. These effects relate to insulin resistance in adipocytes, systemic inflammation and vascular calcification via free radical production, disruption of the intestinal epithelial barrier and alteration of the microbiome^1,6,30^. It is proposed that these molecular changes induced by urea lead to systemic inflammation and cardiovascular damage, major causes of morbidity and mortality in the CKD population^31^. It is important to note, however, that while observational data suggests an adverse impact of urea on clinical outcomes, to the best of our knowledge, controlled clinical studies confirming these clinical effects are currently lacking. These issues will require further investigation before potential targeted therapeutics can be adequately developed.

Future strategies to improve the outcomes of patients with CKD might be directed at more consistent and earlier lowering of serum urea. Interventions that have been proposed include low protein diets, probiotics, prebiotics and modification of the intestinal microbiome^1,31,32^. Clinical trials analysing these therapies have shown promising results in decreasing serum urea and attenuating declines in renal function, but no interventions have thus far been shown to alter cardiovascular or survival outcomes^33^. These studies, however, are limited by sample size and the short length of follow-up. Because many of the proposed biochemical alterations induced by elevated urea are likely to commence prior to the end-stages of CKD it is likely that any effective future therapies will need to be implemented in the early stages of the disease to prevent chronic exposure to elevated urea^1,6,31^.

This study has several limitations. Firstly, it is based on data from a single centre in Australia and the limitations in the generalisability of these results should be noted. However, it was conducted in a large tertiary hospital that shares the typical characteristics of similar centres in resource-rich countries. The single centre-nature of the study also precludes presentation to alternative health services from being included in the 30-day readmission data. Additionally, data on protein intake, hydration status at the time of UCR assessment, BMI and steroid medication use in the study population was not available for data analysis. Future studies should consider incorporating these variables to further investigate how these factors influenced the associations found. Lastly, variation in a patient’s UCR is likely to occurring during an inpatient episode, therefore, the admission UCR may not fully predict clinical outcomes. Despite these limitations, this study provides useful information towards understanding the association between UCR on mortality and morbidity in hospitalised CKD patients. To our knowledge, this is the first study to examine the clinical significance of UCR in non-end stage CKD patients using a large database (> 11,000 patients). As such, it should provide useful information to help clinicians understand the nature of UCR in CKD and guide future research.

## Conclusion

In conclusion, this is the first study to investigate the prevalence, patient characteristics and clinical outcomes of UCR level in a generalised non-end stage CKD population. In this large cohort of CKD inpatients, a UCR level > 100 was common at hospital admission. The admission UCR level also had a potent and statistically significant association with poor clinical outcomes, most notably all-cause inpatient mortality. Further research is required to clarify the relationship underlying changes in UCR level and clinical outcomes, and additional investigations are necessary to determine whether UCR is a potential therapeutic target to reduce the burden of mortality and morbidity in CKD patients.

## Data Availability

The datasets used and/or analysed during the current study are available from the corresponding author on reasonable request.

## Abbreviations

ACEi: Angiotensin-converting enzyme inhibitor
AKI: Acute kidney injury
ARB: Angiotensin II receptor blocker
ATSI: Aboriginal or torres strait islander
CCI: Charlson comorbidity index
CKD: Chronic kidney disease
CKD-EPI: Chronic kidney disease epidemiology collaboration
DARE Centre: The data analytics research and evaluation centre
eGFR: Estimated glomerular filtration rate
ESKD: End-stage kidney disease
GIT: Gastrointestinal tract
ICD-10: International classification of diseases, tenth revision
ICU: Intensive care unit
IQR: Interquartile range
IR: Incidence ratio
KDIGO: Kidney disease improving global outcomes
OR: Odds ratio
SD: Standard deviation
UCR: Urea-to-creatinine ratio
95% CI: 95% confidence interval
